# The Small-RNA Profiles of Almond (*Prunus dulcis* Mill.) Reproductive Tissues in Response to Cold Stress

**DOI:** 10.1371/journal.pone.0156519

**Published:** 2016-06-02

**Authors:** Marzieh Karimi, Farahnaz Ghazanfari, Adeleh Fadaei, Laleh Ahmadi, Behrouz Shiran, Mohammad Rabei, Hossein Fallahi

**Affiliations:** 1 Department of Plant Breeding and Biotechnology, Faculty of Agriculture, Shahrekord University, Shahrekord, P.O.Box 115, Iran; 2 Institute of Biotechnology, Shahrekord University, Shahrekord, P.O.Box 115, Iran; 3 Department of Biology, School of Sciences, Razi University, Bagh-e-Abrisham Kermanshah, Iran; 4 Medical Biology Research Center, Kermanshah University of Medical Sciences, Kermanshah, Iran; University of Balochistan, PAKISTAN

## Abstract

Spring frost is an important environmental stress that threatens the production of Prunus trees. However, little information is available regarding molecular response of these plants to the frost stress. Using high throughput sequencing, this study was conducted to identify differentially expressed miRNAs, both the conserved and the non-conserved ones, in the reproductive tissues of almond tolerant H genotype under cold stress. Analysis of 50 to 58 million raw reads led to identification of 174 unique conserved and 59 novel microRNAs (miRNAs). Differential expression pattern analysis showed that 50 miRNA families were expressed differentially in one or both of almond reproductive tissues (anther and ovary). Out of these 50 miRNA families, 12 and 15 displayed up-regulation and down-regulation, respectively. The distribution of conserved miRNA families indicated that miR482f harbor the highest number of members. Confirmation of miRNAs expression patterns by quantitative real- time PCR (qPCR) was performed in cold tolerant (H genotype) alongside a sensitive variety (Sh12 genotype). Our analysis revealed differential expression for 9 miRNAs in anther and 3 miRNAs in ovary between these two varieties. Target prediction of miRNAs followed by differential expression analysis resulted in identification of 83 target genes, mostly transcription factors. This study comprehensively catalogued expressed miRNAs under different temperatures in two reproductive tissues (anther and ovary). Results of current study and the previous RNA-seq study, which was conducted in the same tissues by our group, provide a unique opportunity to understand the molecular basis of responses of almond to cold stress. The results can also enhance the possibility for gene manipulation to develop cold tolerant plants.

## Introduction

Almond is a perennial species belonging to Prunoideae, a subfamily of Rosaceae. Almond is predominantly self-incompatible. It has sixteen (2*n* = 16) small chromosomes and a small diploid genome of approximately 300 Mbp [1]. Spring frost is one of the most important environmental factors, which limits almond’s production. During winter, buds are dormant and hard. But they swell and expand into blossoms when the temperature rises. As a result, they become less resistant to the cold. Frost can damage 10% to 90% of the almond tree in different phonological stages of flowering [2]. Following our previous study on transcriptome of almond reproductive tissues in response to spring frost [3], we have decided to further expand our understanding of the molecular basis of almond responses to cold stress by conducting small RNA-Seq analysis in the same tissues. As miRNAs are mega bio-regulators in plants [4] and are involved in different stress responses [5, 6], we thought that changes in gene expression in response to cold would also reflect a similar pattern in miRNAs expression pattern.

Plants involvement of miRNAs and their target genes in response to stress conditions is well documented in several studies such as cold signaling regulation [7, 8] and salt and nutrient deficiency [9–11]. For instance, Barakat *et al*. [12] have identified 157 conserved and 230 non-conserved miRNAs sequences in *Prunus persica* in response to cold stress. In *Medicago truncatula*, 283 and 293 miRNAs were identified from control and drought stress libraries, respectively [13]. Zhang *et al*. [14] reported 106 conserved miRNAs from tea leaves treated with cold (*Camellia sinensis*). Sun and colleagues [15] identified 136 known and 68 novel miRNAs in *Raphanus sativus* in a salinity stress experiment. Among these miRNAs, several were characterized as stress responsive. Many studies have shown that miR156/157, miR159/319, miR165/166, miR169, miR172, miR393, miR394, miR396, miR397 and miR398 are up-regulated in response to cold stress [8, 16, 17]. Some of the studies, moreover, have shown the down-regulation of miRNAs such as miR156g-j, miR475a, b and miR476a under cold stress in Populus [18]. Although information regarding miRNAs expression in many species is building up quickly, we do not have any reports regarding the number of miRNAs and their expression pattern in plants such as almond (*Prunus*. *dulcis* (Mill.) D.A.Webb).

Different approaches including forward genetics, direct cloning and bioinformatics prediction followed by experimental validation can identify miRNAs [6, 19]. However, these approaches have limitations such as time, cost and the versatility of the methods. In recent years, next generation sequencing platforms like Genome Analyzer (Illumina Inc.) or Genome Sequencer TM FLX (454 Life Science TM and Roche Applied Science) have been used to detect small RNA molecules and determine the expression levels of both conserved and novel miRNAs [19–22]. In many plant species, this method has been successfully applied to discover miRNAs responses to different abiotic stress. Some of the examples of such applications are the identification of drought stress responsive miRNAs in *Populus trichocarpa* [23], salt stress responsive miRNAs in *Saccharum spontaneum* [24] and heavy metal stress responsive miRNAs in *Medicago truncatula* [25].

Until now, there is no report on the responses of almond’s miRNAs to cold stress, especially in the reproductive tissues, where the cold stress would have the most devastating effects. As the whole genome sequencing of almond is not available, the results of our two studies could be merged to uncover the molecular responses of reproductive tissues of almond to cold stress.

## Results

### Construction and high-throughput sequencing of small RNAs libraries in almond

cDNA libraries of small RNAs were constructed from anther and ovary under cold stress and control conditions in order to identify miRNAs in almond. Our sample collection consisted of anther samples under stress (HSA) and under control (HCA); and ovary samples under stress (HSO) and under control (HCO). All samples were obtained from H gentoptype of almond, which is beleived to be tolerant to cold stress. We have obtained 50 to 58 million raw reads, using Illumina sequencing platform with average read length of 51 nucleotides and the quality of 39 for all samples. GC contents for all samples were 52%, while it was 51% in the HSA.

HCA samples yielded the highest number of small RNA reads of about 58,664,982, while HSO samples contained 50,245,161 reads. 85.74% (12.77% unique reads) from HCA, 78.33% (13.45% unique reads) from HSA, 91.03% (16.09% unique reads) from HCO and 88.85% (17.77% unique reads) from HSO were obtained after filtering. The adaptors and all contaminanted small RNAs including tRNA, snRNA, snoRNA and rRNA were removed and residual reads were queried against known miRNAs in miRBase (version 20). 2,137,645, 1,626,754, 2,767,683 and 2,197,689 reads from HCA, HSA, HCO and HSO libraries were identified as conserved miRNAs. The rest of the sequences were considered as non-conserved sRNA sequences.

### Identification of conserved miRNAs in almond

**Fig 1:**
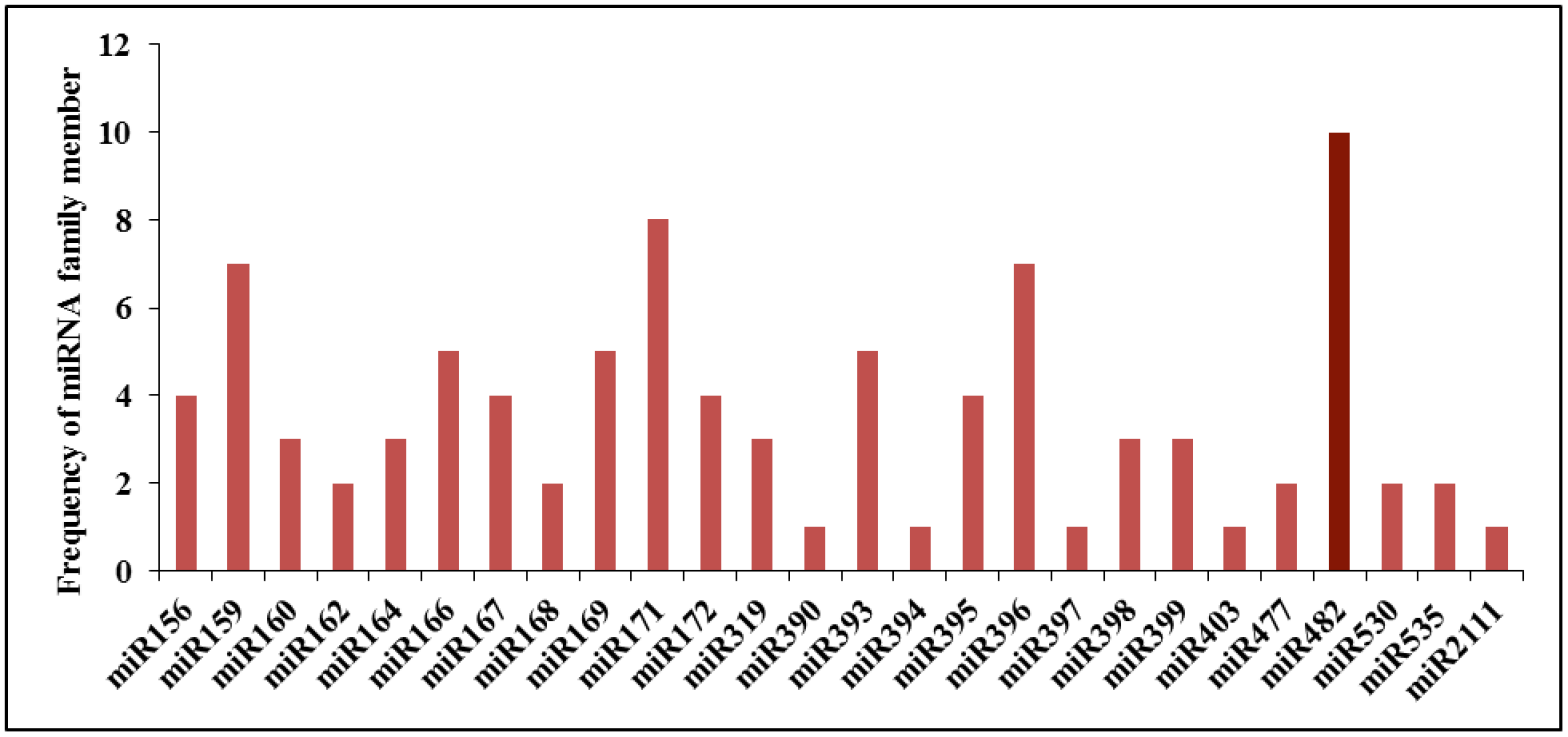
The distribution of highly conserved miRNAs family size in *prunus dulcis*.

**Fig 2:**
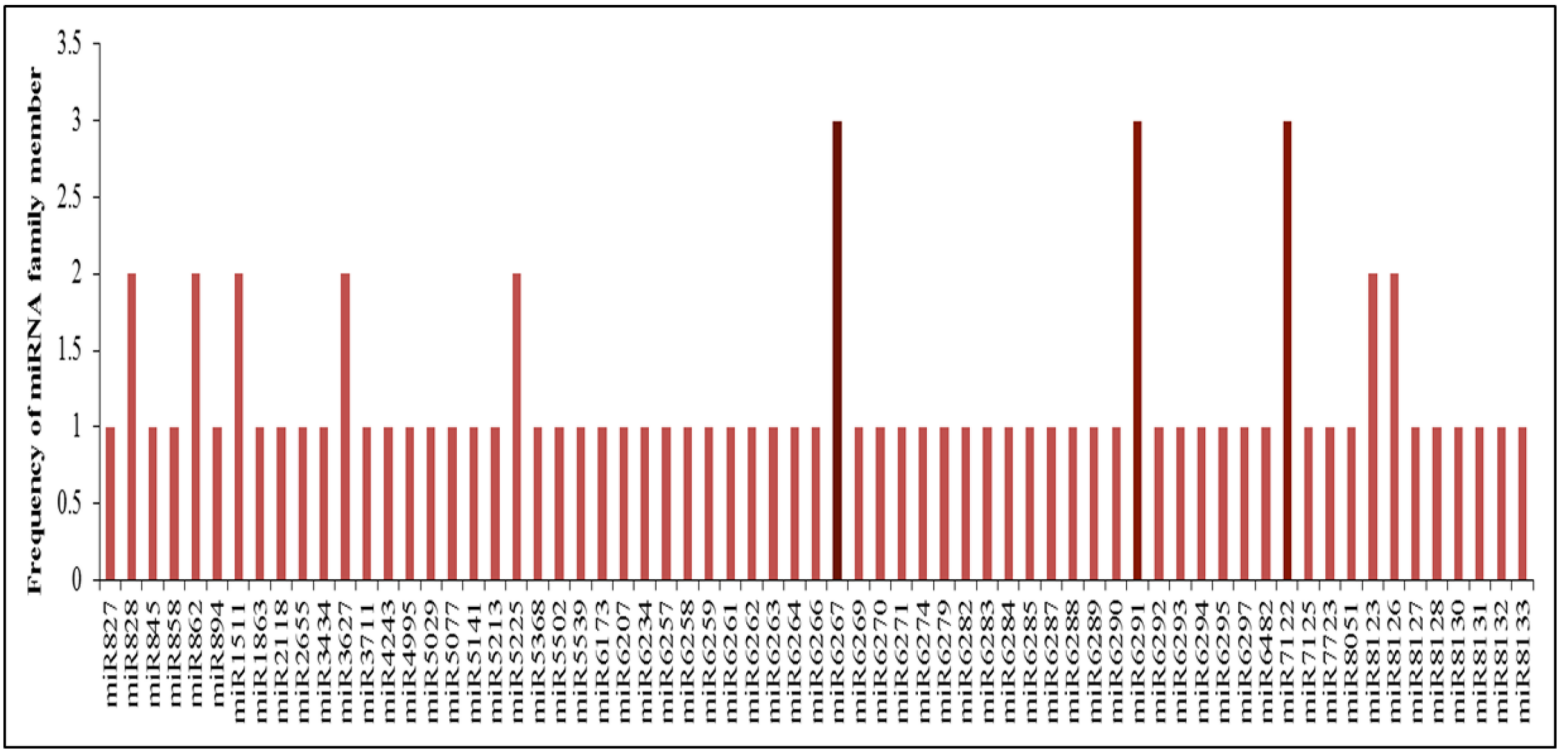
The distribution of conserved miRNAs family in *prunus dulcis*.

After performing a blast against miRBase database (version 20), sequence similarity releaved the presence of 131, 122, 123 and 119 miRNAs in the HCA, HCO, HSA, and HSO libraries, where 94 miRNAs were unique. Out of these 94 uniquly identified conserved miRNA families in four librarires, 26 were highly conserved and 68 were conserved in some plant species (S1 Table). The highly conserved miRNA families contained 1–10 members in our dataset. miR482f family had the highest number of members (10 members). This was followed by miRNA 171 family, having 8 members (Fig 1). On the other hand, among the conserved miRNAs family, miR6267, miR6291, miR7122 with 3 members had the highest number of members, while the most of the other conserved miRNA families conatined only one member (Fig 2).

**Fig 3:**
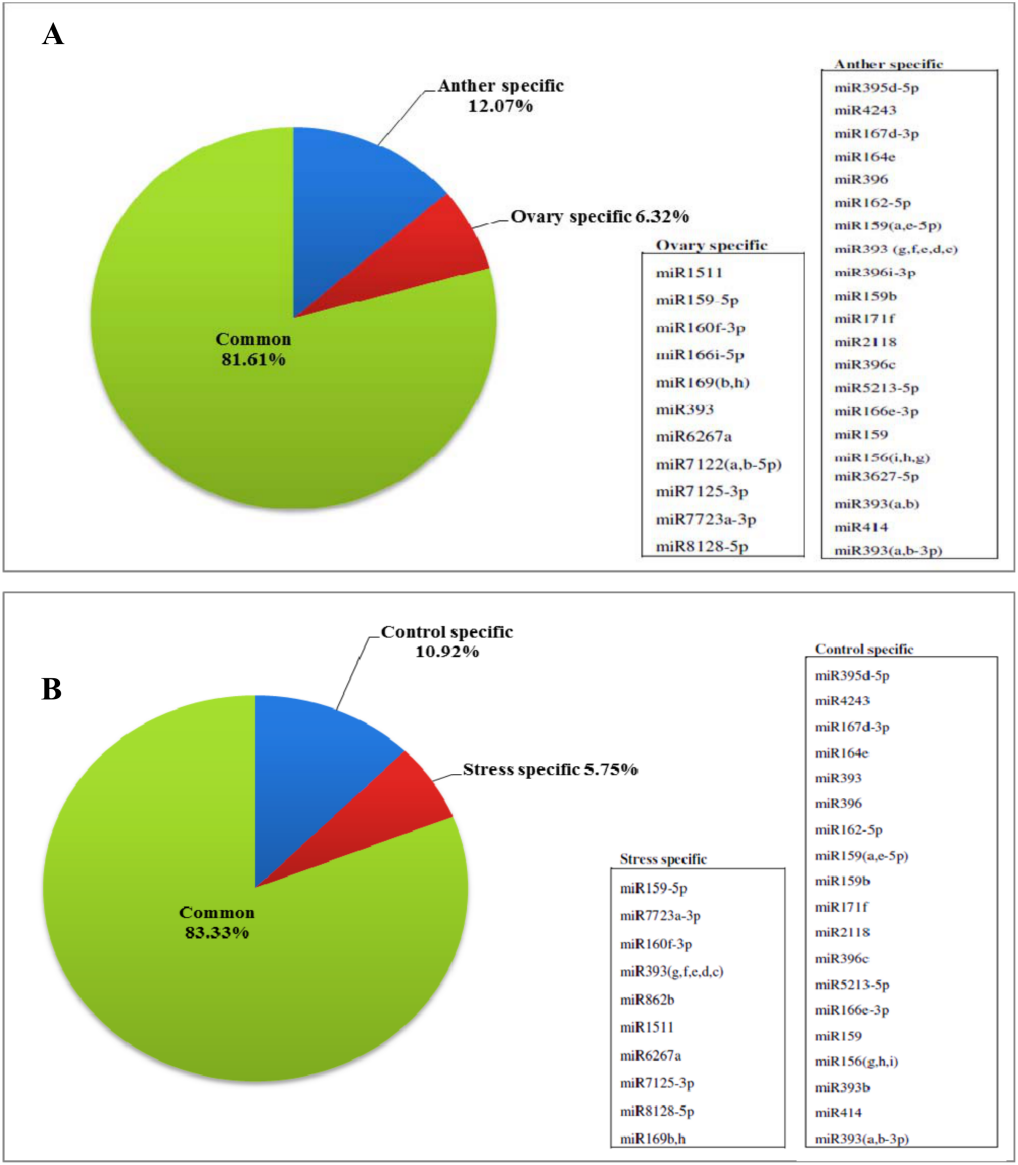
Common and specific miRNA families identified by high throughput sequencing. A: Common and specific miRNAs in reproductive tissues of almond; B: Common and specific identified miRNAs under control and cold stress.

**Fig 4:**
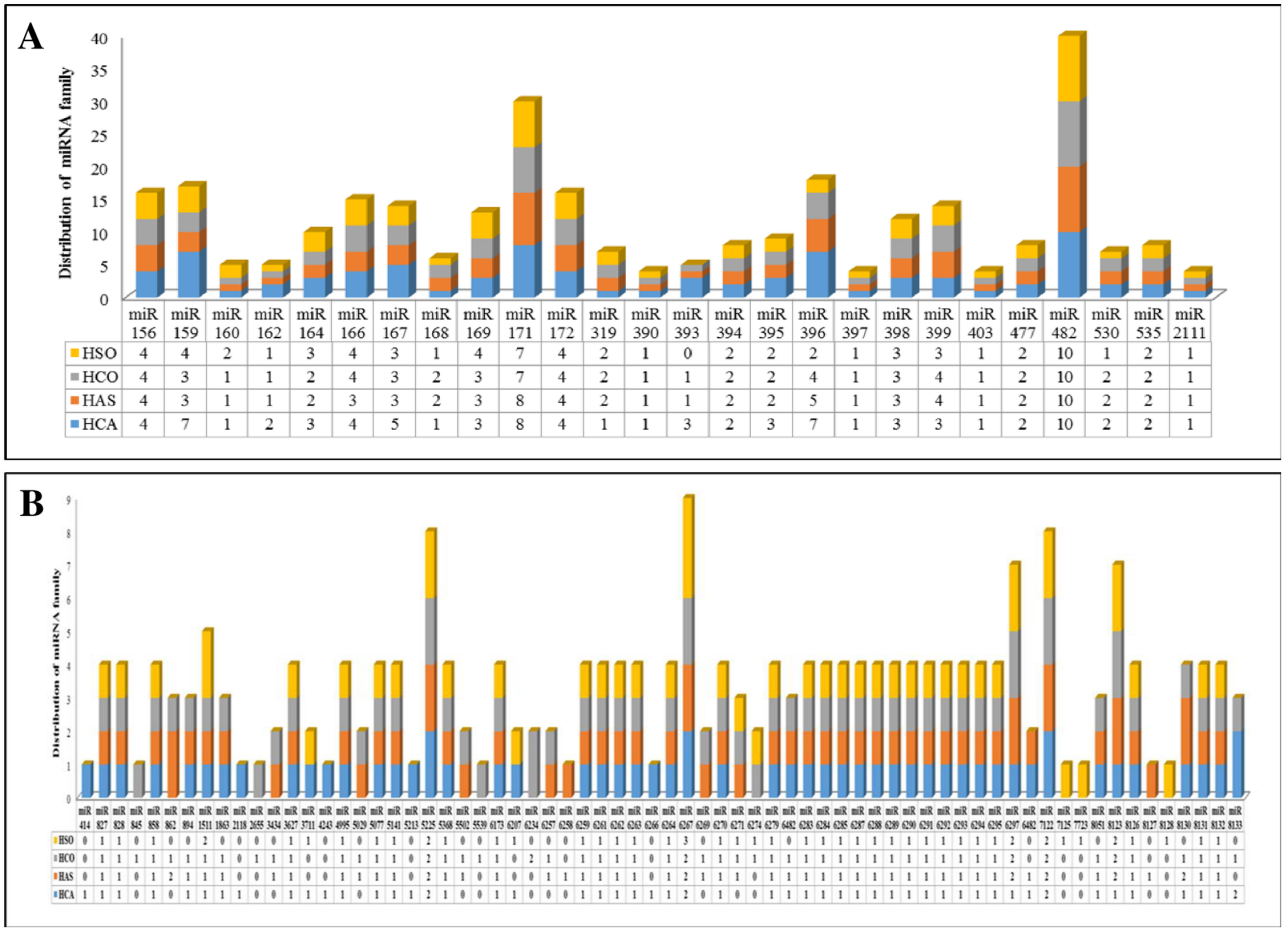
The distribution of known miRNA families in HCA, HSA, HCO and HSO libraries. A: Highly conserved; B: Conserved in some species.

A comparison between miRNA family members in these four libraries showed that 32 miRNA families had tissues-specific expression pattern. Among these miRNAs, 21 were expressed only in the anther and 11 in the ovary (Fig 3A). Interstingley, 10 miRNAs found to be expressed solely under cold-stress conditions. Nineteen miRNAs were specifically expressed under control conditions and therfeore, their expression was not detectable under stress conditions (Fig 3B). Also the size distribution of miRNA families between the four libraries revealed 62 common miRNAs, though the number of members were different in each library (Fig 4A and 4B).

### Identification of novel miRNAs in almond

**Fig 5:**
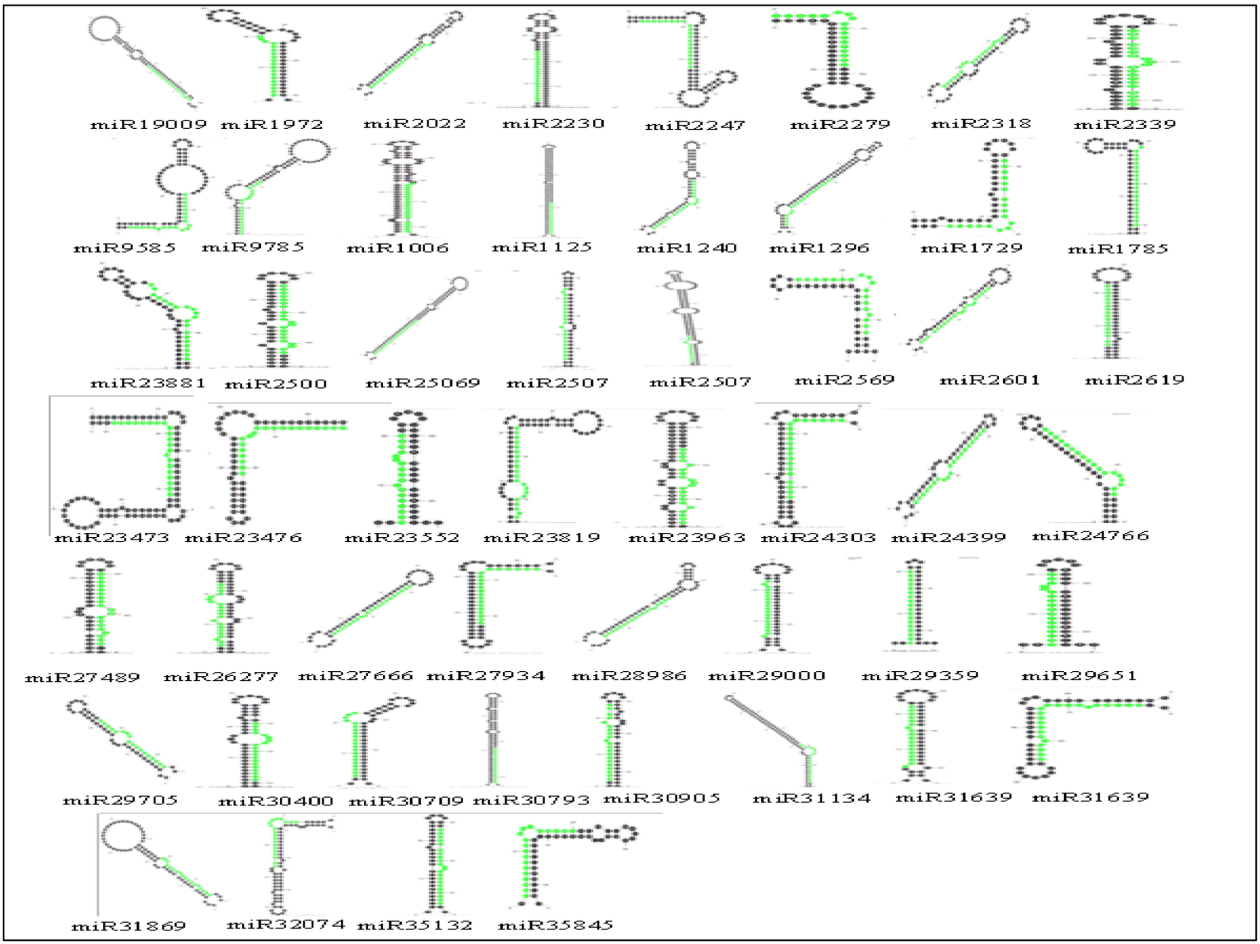
The structure of candidate novel miRNAs precursor.

In our study, we found many reads without any hits in the RNAs datasets. They were 289,302, 245,497, 151,928 and 177,393 reads in the HCA, HSA, HCO and HSO libraries, respectively. Therefore, these reads were mined to identify non-conserved miRNAs. Following miRNA annotation criteria set by Meyer and collaborators [26], we successfully identified 59 novel miRNAs sequences. The stem-loop structures of these miRNA precursors were obtained by Mfold [27] and are presented in Fig 5. The mauture length of these miRNAs varied between 18 and 22 nt. The most abundant length was 21 nt. The precursor lengths were found to be between 45 to 147 nt. The minimum free energy (MFE) varied from -71.8 to -8.5 kcal/mol (S2 Table). The MFE index (MFEI) for these precursers, as the most important criterion for prediction of stem-loop structure of miRNA [28], were calculated according to Zhang *et al*. [14]. Notabley, the mature sequences of 34 novel miRNAs were located on 3' arm of their precursers and the rest were located on 5' arm.

### Expression pattern analysis of known miRNAs

**Fig 6:**
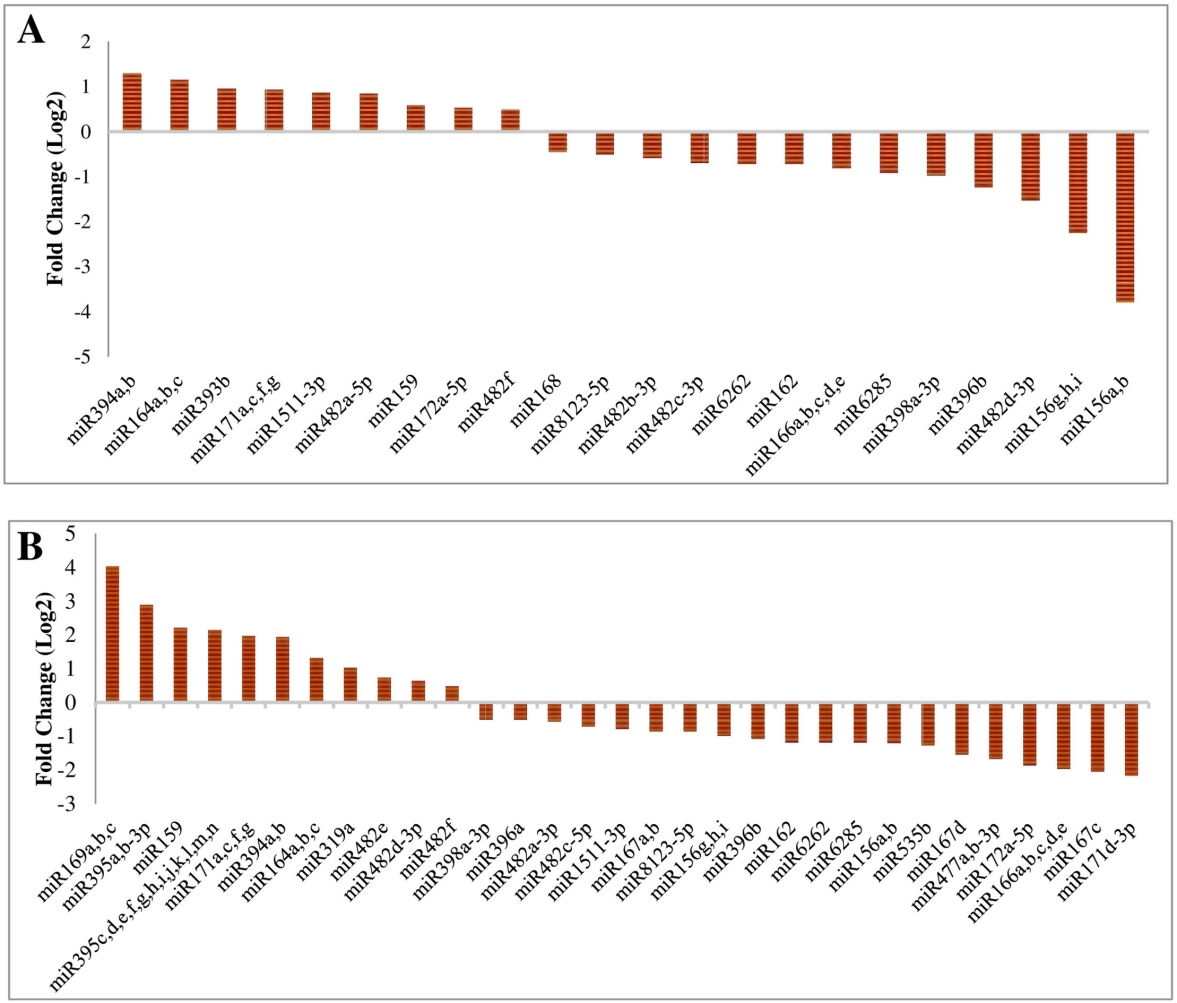
Relative differentially expressed conserved miRNA in reproductive tissues. A: Anther; B: Ovary.

Expression analysis of conserved miRNAs indicated that miR159, miR166, miR482 and miR1511 families had the highest expression in the four libraries. However, distinct expression variation was observed within the members of some families. For instance, in HCA library miR482f had 109668 copies, whereas the read number for miR482b-5p was only 10. The expression pattern of miRNAs in anther indicated that 53 miRNAs had higher expression in control conditions compared to ovary. In contrast, 48 miRNAs were up-regulated under stress conditions in anther compared to ovary. Moreover, 41 miRNAs showed similar pattern in both tissues exposed to cold stress. Therefore, we suggest that these miRNAs should be considered as cold stress responsive miRNAs. From these miRNAs, 22 were down-regulated and 19 were up- regulated. Among differntially expressed miRNAs, miR482d-3p was up-regulated in anther, while it was down-regulated in ovary. In contrast, miR172a-5p and miR1511-3p were up- regulated in ovary and down-regulated in anther (S3 Table). Comparative analysis of expression pattern in these two reproductive tissues revealed 11 and 9 up-regulated miRNAs in anther and ovary, respectively (Fig 6A). Additionally, 20 and 13 down-regulated miRNAs were found in these tissues respectively (Fig 6B). Futhermore, the analysis identified 5 up-regulated and 11 down-regulated miRNAs in anther with no significant changes in ovary. However, we found 8 repressed miRNAs in anther, while their expression was iduced in ovary tissues.

### Expression pattern validation of miRNAs in reproductive tissues of almond

**Fig 7:**
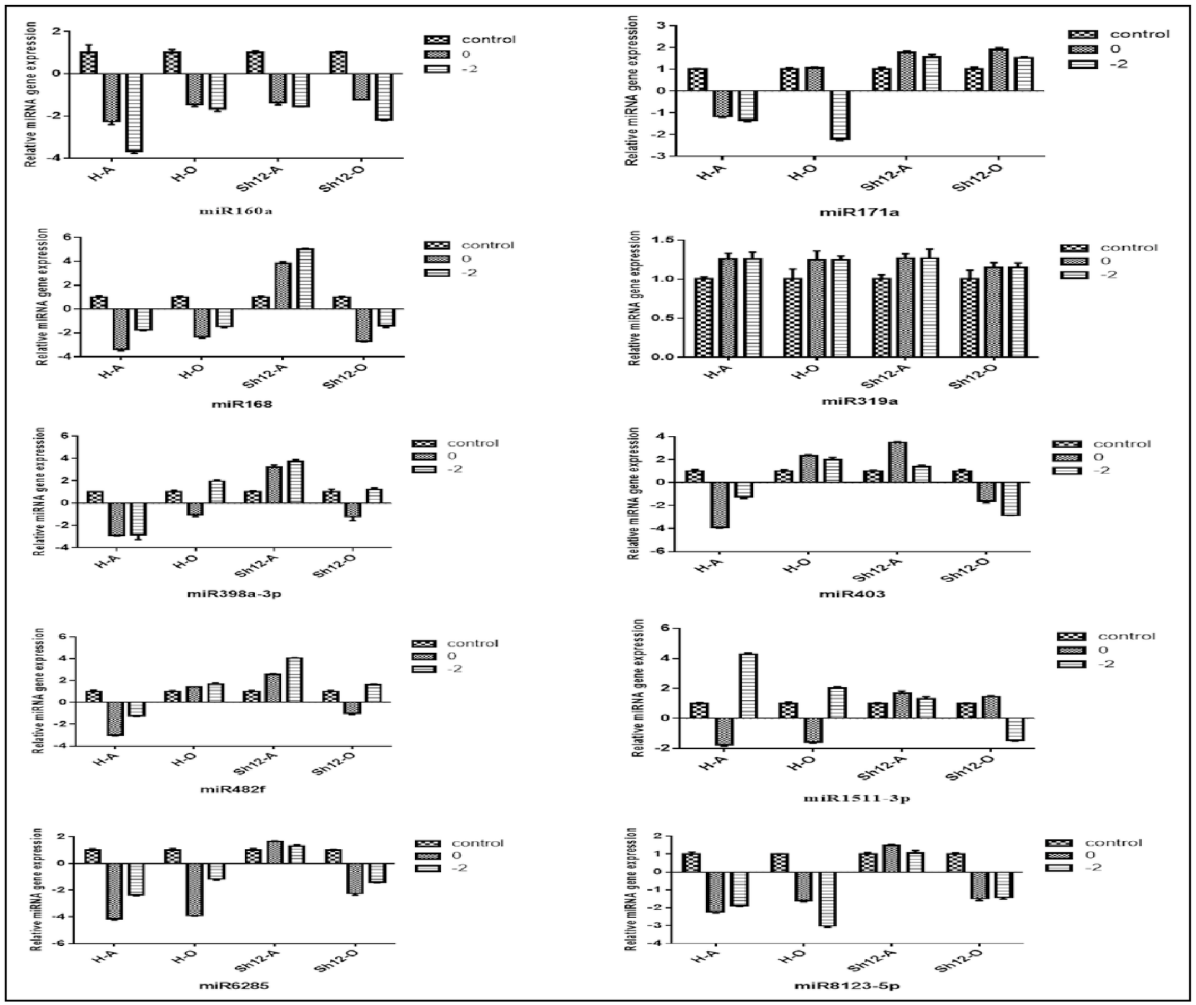
qPCR analysis for validation of differentially expressed miRNAs expression in reproductive tissues of almond. Each bar represented the mean ± SE.

**Fig 8:**
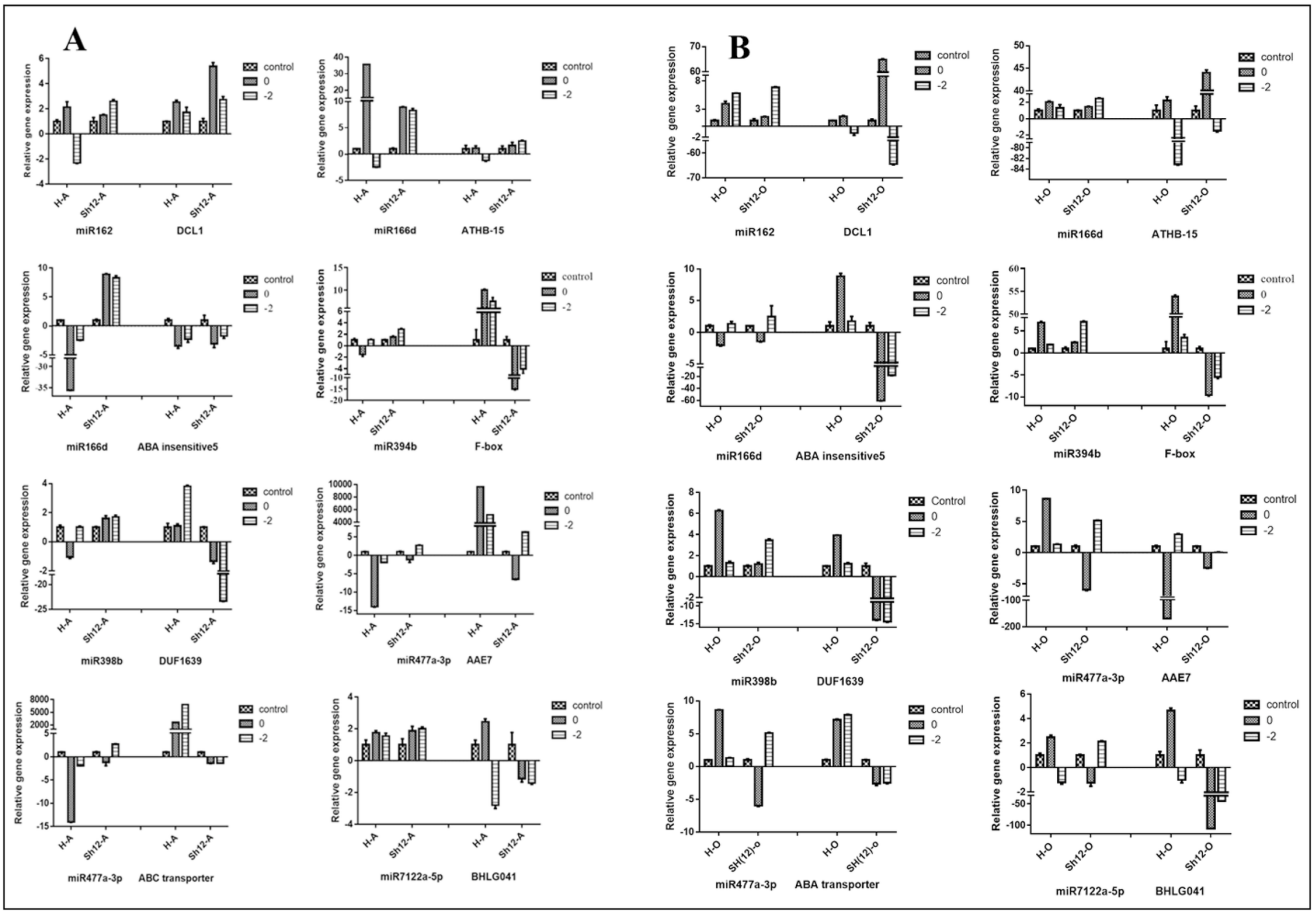
qPCR analysis of cold stress-responsive miRNAs and their targets expression profiles. A: Anther; B: Ovary. Each bar represented the mean ± SE.

To validate the expression patterns detected for miRNAs in the high throughput sequencing, 16 differentially expressed miRNAs were selected and analyzed with qPCR. In addition, to further investigate the miRNA responses to cold stress, we included a rather cold-sensitive genotype (Sh12) in our analysis. Expression pattern comparison between small RNA sequencing data and qPCR results indicated very similar patterns of expression for 11 and 7 miRNAs in anther and ovary samples under -2°C treatment, respectively. In anther, furthermore, miR162, miR166d, miR168, miR171a, miR398a-3p, miR403, miR482f, miR6285 and miR8123-5p were expressed differentially in H genotype and Sh12 varieties. However, in ovary, miR403, miR1511-3p and miR7122a-3p displayed differential expression in these two genotypes. On the other hand, miR7122a-3p in anther and miR162, miR166d, miR398b, miR398a-3p, miR160a, miR319a, miR168, miR6285 and miR8123-5p in ovary had similar expression pattern in both tolerant and sensitive varieties. Unsurprisingly, some miRNAs displayed different mode of expression in anther and ovary samples of these two almond verities. For example, miR162, miR477a-3p and miR482f in H genotype and miR168, miR403, miR6285 and miR8123-5p in Sh12 showed different expression between these studied tissues. (Figs 7, 8A and 8B). Among all 16 differentially expressed miRNAs studied by qPCR, cold stress repressed the expression of 10 miRNAs in anther tissue of H genotype and had no effects on the expression of miR160a, miR319a, miR394b, miR398b, miR1511-3p and miR7122a-5p. In contrast, miR162, miR171a, miR319a, miR394b, miR398b were up-regulated in Sh12 under cold stress. In addition, it was found that -2°C cold treatment induced the expression of miR398b, miR477a-3p, miR394b, miR7122a-3p, miR162 and miR166d in both ovary and anther tissues of Sh12 variety. Different expression pattern were detected for some miRNAs including miR1511-3p, miR398a-3p, miR166d and miR7122a-5p in these varieties treated with two temperatures. For instance the expression of miR1511-3p, miR398a-3p and miR166d was lower in 0°C compared to that of -2°C. Transcript of miR394b and miR398b was not altered in the anther tissue of H genotype in -2°C. But the two miRNAs were down-regulated in 0°C. Among the tested miRNAs, miR477a-3p, which increased 8.6- fold in the ovary tissue of H genotype under 0°C, and miR166d, with 8.27- fold increase in Sh12 under -2°C, showed the most changes in their expression pattern. In anther tissue of H genotype, miR166d and miR160a had the greatest reduction of expression under 0 and -2°C, respectively. MiR319a was the only miRNA, which was up-regulated in both varieties under both stress conditions. In contrast, miR160a was the only miRNA that was down-regulated under cold stress in both varieties. In other studies this miRNA was introduced as a cold-induced miRNA [29].

In this study, among the predicted targets for cold-responsive miRNAs (S4 Table), the expression pattern of 6 miRNA's targets namely *DUF1639* (miR398b target), ABC transporter and *AAE7* (miR477a-3p targets), *FBX* (miR394b target), *BHLG041* (miR7122a-3p target), *DCL1* (miR162 target) and *ATHB-15* and ABA-insensitive 5 (miR166d targets) were examined in the lab. Negative correlation between some of these miRNAs and related targets was detected (Table 1). For instance, cold-stress repressed miR477a-3p expression was negatively correlated with the conversely up-regulated expression of both ABC transporter and *AAE7* genes in anther tissue of H genotype. On the other hand, the up-regulation of miR394b was followed by the down-regulation of the expression of *FBX* gene in Sh12. Among the studied targets, ABC transporter gene, one of the miR477a-3p targets, showed the highest number of fold changes in anther tissues of cold-tolerant genotype H.

**Table 1:** Expression profile between miRNA and corresponding targets.

	cold-stressed mode								
miRNA-target	Tm (°C)	H				SH (12)			
		anther		ovary		anther		ovary	
		miRNA	Target	miRNA	Target	miRNA	Target	miRNA	Target
miR162- *DCL1*	0	down	up	up	down	up	up	up	down
miR166d-ABA INSENSITIVE 5		down	down	down	up	up	down	down	down
miR166d- *ATHB-15*		down	up	down	down	up	up	down	down
miR394b- *FBX*		down	up	up	up	up	down	up	down
miR398b- *DUF1639*		down	up	up	up	up	down	up	down
miR477a-3p- ABC transporter		down	up	up	up	down	down	down	down
miR477a-3p- *AAE7*		down	up	up	up	down	down	down	down
miR7122a-3p- *BHLG041*		up	up	up	down	up	down	down	down
miR162- *DCL1*	-2	down	up	up	down	up	up	up	down
miR166d- ABA-INSENSITIVE 5		down	down	up	up	up	down	up	down
miR166d- *ATHB-15*		down	down	up	down	up	up	up	down
miR394b- *FBX*		up	up	up	up	up	down	up	down
miR398b- *DUF1639*		no change	up	up	up	up	down	up	down
miR477a-3p- ABC transporter		down	up	up	up	up	down	up	down
miR477a-3p- *AAE7*		down	up	up	up	up	up	up	up
miR7122a-3p- *BHLG041*		up	down	down	down	up	down	up	down

### Identification of miRNA-guided cleavage of target mRNA using RLM-RACE

**Fig 9:**
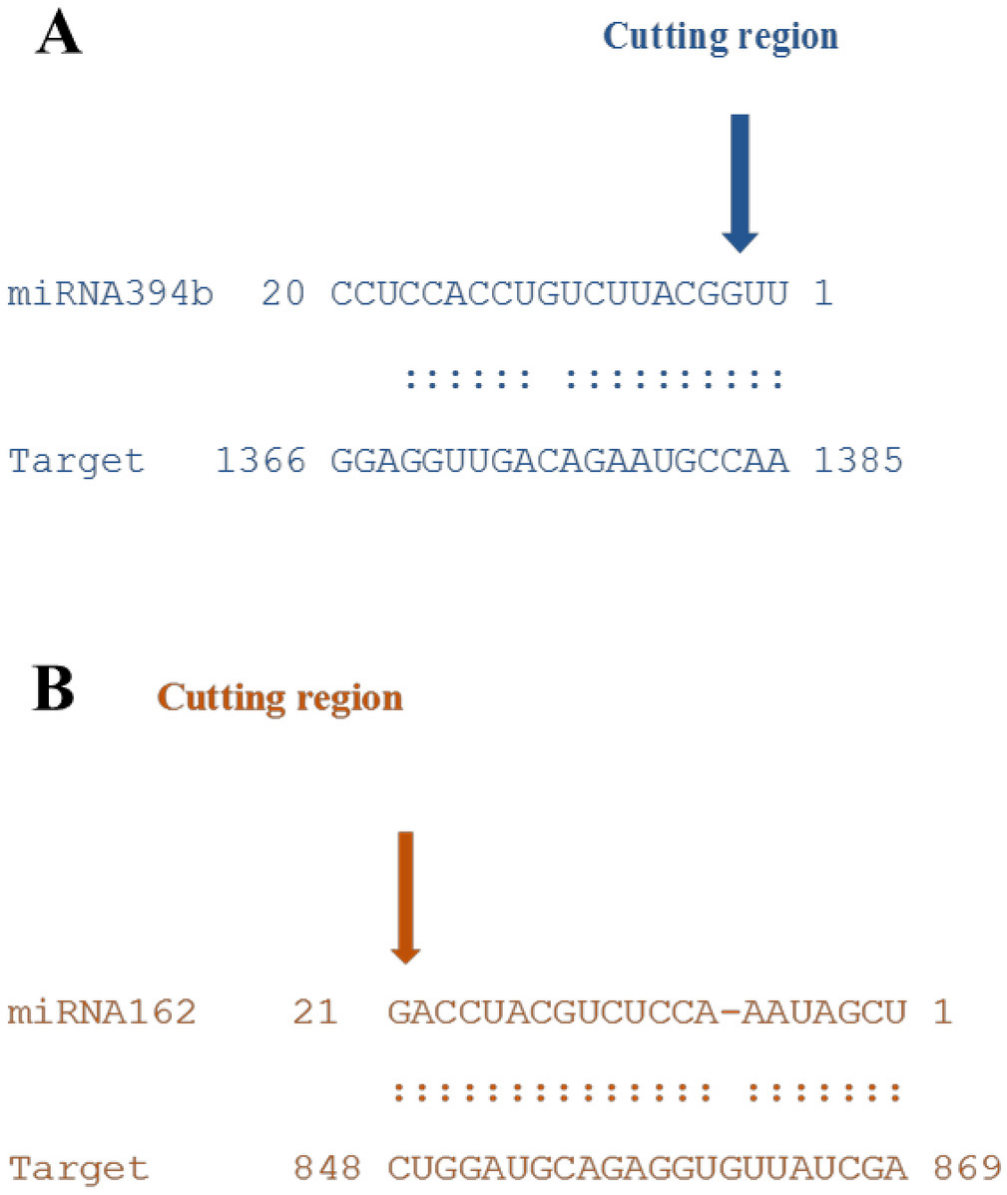
Experimental validation of predicted miRNA targets. Bottom strands depict target mRNA sequences. A: *FBX*; B: *DCL1*. Top strands depict the miR394b and miR162. Matches (Watson—Crick pairing) are indicated with vertical dashes, mismatches are unmarked. Arrows indicate cleavage sites verified by 5′ RACE assay.

In order to corroborate whether *FBX* and *DCL1* genes are direct targets of repression by miRNA394b and miRNA162, through miRNA-directed cleavage of their mRNA, we used the modified 5′ RACE technique for mapping the cleavage sites of miRNA on these two genes transcripts. We used RNA from plants treated for 3 h at 0°C. Cleavage of the miR394b target (*FBX*), and miR162 target (*DCL1*) was confirmed by 5′ RACE assay. PCR RACE products were purified and sequenced. The results indicated that the site of cleavage is located downstream of the second nucleotide from the 5′ end of the *FBX* mRNA (Fig 9A) and the second nucleotide from the 3′ end of the *DCL1* mRNA (Fig 9B). These findings confirmed that *FBX* gene and *DCL1* gene were directly targeted for cleavage by miR394b and miR162, respectively.

## Discussion

Like other abiotic stresses, cold stress threatens many plant species. Most organisms have an optimum temperature for appropriate growth and development. Thermal fluctuations beyond the threshold level lead to stress [30]. Plants respond to cold stress through specific mechanisms that involve reprogramming gene expression. The important role of miRNAs in response to different environmental stresses has been verified in previous studies [21, 15, 31–35]. Several works have introduced cold stress responsive miRNAs in different plant species such as Arabidopsis [7, 16], tea [14], tomato [36, 37], rice [38], wheat [39, 40], peach [12], *Populus* [41] and *Brachypodium* [6]. No study has been done on the miRNAs regulatory response to cold stress in almond. This study, therefore, was conducted to identify the cold responsive miRNAs and their targets in almond.

### Identification of conserved and novel miRNAs in almond

High throughput sequencing analysis of sRNA libraries in almond's reproductive tissues under two thermal treatments (10 and -2°C) led to identification of 94 conserved miRNA families and 59 novel miRNAs. Size distribution of conserved miRNAs showed that miR482, miR171, miR159 and miR396 families have the highest number of miRNAs. According to, the study of Barakat *et al*. [12], however, miR395 and miR169 were represented most frequently in peach. The comparison between known miRNA families in some Rosaceae species revealed that 28 miRNAs were specific to Prunus tree species. This result can potentially show the roles of these miRNAs in initiation of specific characterization in this species.

### The distribution of miRNA family size in some *Rosaceae* species

**Fig 10:**
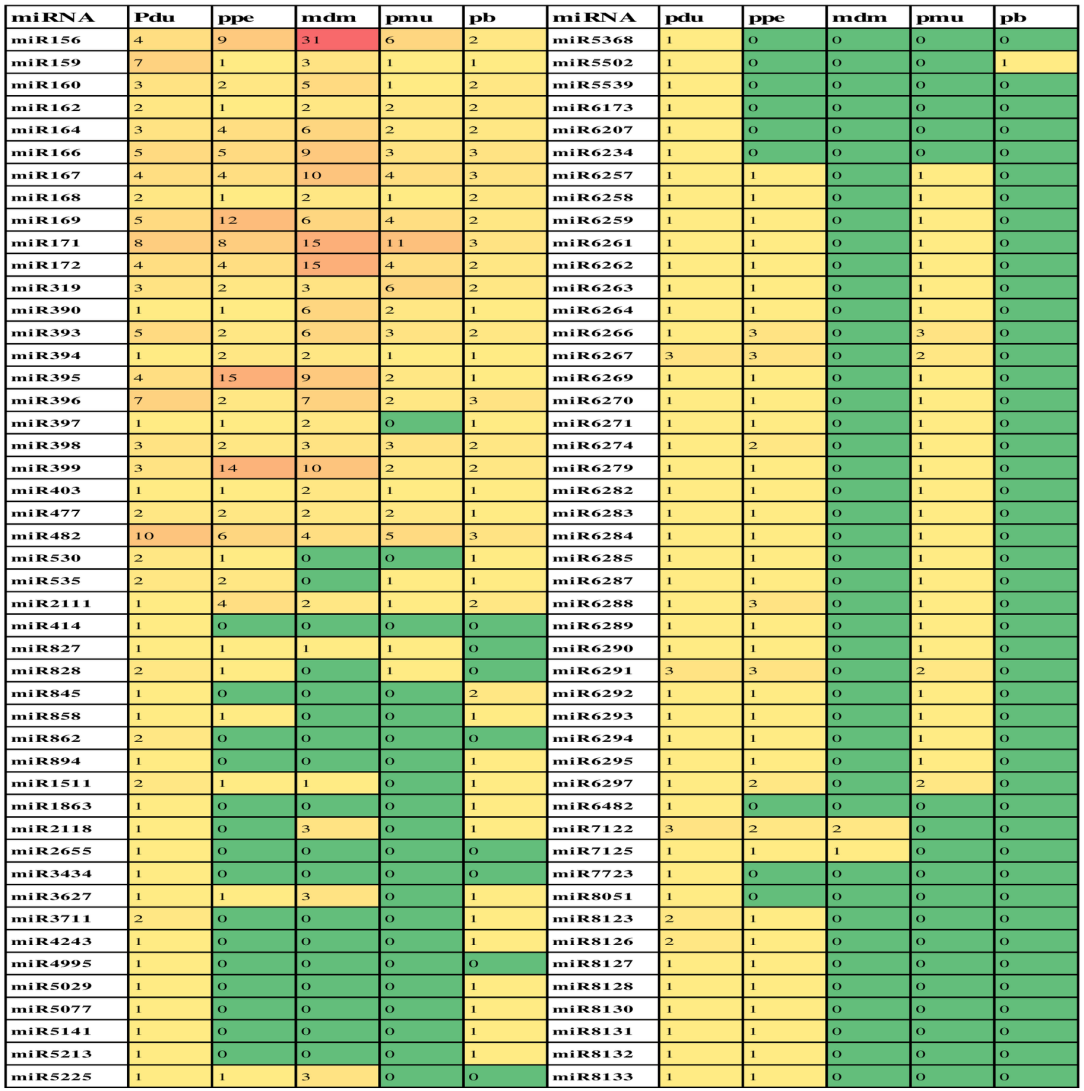
Comparison of known miRNAs across almond (pdu) and other tree species of Rosaceae family. Peach (ppe), Apple (mdm), Japanese apricot (pmu), and Pear (pb).

The number of memebers in miRNA families of four Rosaceae tree species, including *Prunus persica*, *Malus domestica*, *Prunus mume*and, and *Pyrus bretschneideri*, were compared with those of *Prunus dulcis*. Almond and peach shared 70 known conserved miRNA families, indicating a remarkable homology. Approximately, all highly conserved miRNAs are present in these species, except miR 535 and miR530 that are only presnt in apple and miR 397 and miR 530 that are specific to Japanese apricot. Among the miRNA families stduied, miR156 (with 31 members) was the largest family in *Malus domestica*. The size distribution of other groups of known miRNAs showed that some of them are shared only among almond, peach and apricot. This group of shared miRNAs includes miR6257, miR6258, miR6259, miR6261, miR6262, miR6263, miR6264, miR6266, miR6267, miR6269, miR6270, miR6271, miR6274, miR6279, miR6282, miR6283, miR6284, miR6285, miR6287, miR6288, miR6289, miR6290, miR6291, miR6292, miR6293, miR6294, miR6295 and miR6297. That is why Gao et al. [42] considered these miRNAs as unique to *Prunus* plants. Interstingley, 10 miRNA families including miR414, miR862, miR2655, miR3434, miR4995, miR5368, miR5539, miR6173, miR6207 and miR6234 did not have any orthologous in other species. As a result, they were considered to be specific to almond (Fig 10).

### Identification of cold-responsive miRNAs in almond

The miRNAs that were identified with differential expression in the current study have different roles in plant molecular biology. Various studies have shown that miR156, miR163, miR169, miR172, miR398 and miR399 play important role in flowering time. They are also introduced as temperature-responsive miRNAs [17]. MiRNAs, including miR172 and miR156, affect the development of the plant by controlling *AP2* (floral patterning genes) and *SPL* (squamosal promoter binding like). These miRNAs have regulatory roles on transition from juvenile to the reproductive phase [43]. MiR156-*SPL* and miR172- *SVP* (short vegetative phase) make up the regulatory circuit and are known as flowering-time regulators in response to ambient temperature. Using these regulatory mechanisms, plants can adapt their development with temperature fluctuation [17]. MiR159 targets the gibberelic acid MYB (*GAMBY*) gene and regulates seed germination and the formation of the anther [29]. Some of the identified miRNAs have regulatory roles on other miRNA biosynthesis. For example, miR162 effects the regulation of *DCL1* (dicer-like 1) [44], and miR171, which in turn controls *SCR* gene (Scarecrow-like protein). In its own turn, SCR protein, in cooperation with *SHR*, activates the expression of miR165a and miR166b [29]. Notably, some of the miRNAs are mainly involved in regulating stress-responsive genes. For instance, it was revealed that miR160 is a cold-induced miRNA [29]. Or it was found that miR169, the main regulator of NFYA1 (Nuclear transcription factor), displayed different expression pattern in response to drought, salinity, low temperature, etc. In addition, miR7122a-3p gets involved in response to environmental tensions of plants by targeting *BHLG041* gene as part of replication elements [45]. In study of Alisoltani *et al* [46], this miRNA was reported as a positive regulator under cold stress. In this investigation, miR7122a-3p/HOS1 was introduced the only post-transcriptional regulator in gene regulatory network in almond in response to cold stress. MiR166 regulates several genes such as *ATHB*, *REV* and ABA insensitive 5, is involved in stress-response of plants to different conditions. In soybean, for example, miR166b was up-regulated under drought, salinity, and alkalinity [47], while miR166a-5P and miR166f were down-regulated under abiotic stress [48]. This variable regulatory pattern revealed different function of miRNAs from the same family [14].

By using high throughput sequencing, this study identified 12 up-regulated miRNAs (miR159, miR164a/b/c, miR171a/c/g/f, miR394a/b, miR482f and miR7122a-5p) and 15 down-regulated miRNAs (miR156a/b, miR162, miR166a/b/c/d/e, miR396b, miR398a-3p, miR6262 and miR8123-5p) that respond to cold stress in both anther and ovary tissues of almond.

The induction of miR169, miR393 and miR394 and miR398 in almond under cold stress is in agreement with findings in Arabidopsis [7, 8, 16]. The repression of miR482 in the anther tissues of H genotype was consistent with the findings about tomato [49]. On the other hand, the induction of miR166 and miR396 in Arabidopsis and the induction of miR477 in Populus are in contrast to the results about almond [7, 41]. The differences in the expression of these miRNAs among different studies may be due to differences in species, cultivars (genotypes), and the extent and duration of cold stress in different studies [50].

Among the miRNAs, which are involved in cold stress response, miR319a is known as positive regulator by high throughput sequencing and experimental confirmation. Up-regulation of miR319a under both cold stress treatments in the two studied varieties was observed. Other studies have also noted the over-expression of miR319 under cold stress treatments [29, 51, 52]. The members of this miRNA family are usually up-regulated when plants are exposed to different stresses [53]. MiR319a is the first plant miRNA that was identified by forward genetics. This miRNA regulates TCP family, especially *TCP4*. This transcription factor is involved in the development of leaf and flower, mostly the petal and stamen [29, 53–55]. The other predicted target (*GAMYB*) regulates the growth of the plant. Zhou *et al*. [56] has demonstrated that the enhanced abiotic stress tolerance in *Agrostis stolonifera* is the result of the overexpression of osa-miR319a and consequently the significant down-regulation of its target gene. Therefore, previous studies and our own results imply that miR319a could be the main target for engineering tolerant plants to abiotic stress.

It is well known that the genotypes of different plant species affect their response to the same abiotic stress. To test this point, the expression pattern of 16 candidate miRNAs was investigated in a sensitive and a tolerant variety under cold stress treatments. Among the studied miRNAs, miR162, miR166d, miR168, miR171a, miR398a-3p, miR403, miR482f, miR6285 and miR8123a-5p in anther and miR403, miR1511-3p and miR7122a-3p in ovary appeared with differential expression in H genotype and Sh12. With this result on the expression pattern of miRNAs and their targets, positive and negative regulators under cold stress could be identified in almond. Such results could then be further integrated into more sophisticated genetic engineering approaches to develop tolerant plants. Among the miRNAs, miR6285 and miR8123a-3p have more potential to be considered as specific cold-responsive miRNAs in almond. Interestingly, these two miRNAs were also identified as specific miRNAs in Prunus species.

### Assessment of cold stress response mediated by regulatory roles of studied miRNAs

When plants encounter different kinds of stresses, responsive genes are expressed. These stress-responsive genes could be categorized into two groups. The first group includes stress tolerance inducer proteins such as sugar transporter, which is predicted for miR394b in this study. The second group mostly contains transcription factors, which regulate signal transduction pathways. Function analysis of the second group can provide useful information on the regulatory gene networks involved in cold stress response [57]. In this investigation the majority of the predicted targets for the altered miRNAs belong to the second group. For example it was found that miR172a-5p and miR169a regulates two transcription factors, namely APETALA2 protein and nuclear transcription factor Y (subunit A-1).

Furthermore, the expression patterns of target genes of 6 miRNAs were studied. One of these genes, *ATHB* (homeodomain leucine zipper) was predicted to be miR166d target. In a study by Kim *et al*. [58] on hot pepper, the induction of *ATHB* and CaCBFIB (as dehydration-responsive element) under low temperature stress (4°C) and their interaction during cold stress have been discovered. Kim *et al*. [58] consequently identified *ATHB* gene as an important regulator of dehydration-responsive element. Different studies have shown the overexpression of dehydration-responsive elements followed by the induction of cold-responsive genes, which leads to the inhibition of cellular dehydration and increase in freezing-stress tolerance [57]. These results indicate that exposure of plants to cold stress down-regulates miR166 and, subsequently, increase the expression of *ATHB* gene. In the almond the negative regulatory role of miR166d on *ATHB*, which led to overexpression of this gene, was only observed in the anther of the H genotype under 0°C, while in other treatments such negative correlation was not detected.

The other cold-induced miRNA in almond was miR398b; and *DUF1639* was predicted to be its target. In addition to *DUF1639*, miR398 controls the expression of at least four target genes. Many of DUFs (Domains of unknown function) are highly conserved in plants, indicating their important biological roles. For example Bischoff *et al*. [59] showed TBL role (a DUF protein) in synthesis and deposition of secondary wall cellulose. Moreover, Mawlong *et al*. [60] suggested the important roles of DUF sequence, which is present in AP2/ERF-N22 (2) (Apetela type 2 transcription factor/ ethylene responsive factor) in stress tolerance, plant growth and its development. It is believed that DUFs or PUFs (proteins of unknown function) are required in special conditions [61] such as cold stress. Different studies displayed the importance of DUFs in plants’ response to stress. For instance one DUF named *Esk1* has been recognized with negative regulatory function in cold acclimation [60]. In another study, mutation in *ESKIMO1* (a DUF protein) induced freezing tolerance in Arabidopsis [62].

In this study the induction of miR394b in cold stress (0°C and -2°C) caused repression of *FBX* gene expression in both the reproductive tissues of Sh12 variety. In other words, the negative regulatory role of miR394b on the production of F-box protein was only observed in Sh12. The overexpression of miR394b in response to cold stress in almond was similar to those of Arabidopsis, tobacco, rice and poplar [16; 63, 64]. But the over-expression of an F-box protein gene in rice caused a reduction in abiotic stress tolerance and the growth of the root. From these results one can confer that the mechanism of plants’ response to abiotic stresses is through the induction of miR394b and the repression of F-box protein. Further, the role of miR394 in direct response to multiple stresses has been proved [6]. In a study by Huang and collaborates [65], miR394 has been shown as a versatile miRNA that play important role in various stresses. A large gene family in plants encodes F-box proteins. In Arabidopsis, 700 different F-box proteins were recognized, but the function of many of them has not been investigated [66]. These proteins are involved in a large number of biological processes such as self-incompatibility, leaf senescence, branching and response to different biotic and abiotic stress [67]. The proteins are also involved in abiotic stress response through ubiquitin pathway [68]. One of the reasons a negative correlation has not been observed between the expression of miR394b and its target in the H genotype might be the lag in the response to the stress. Other reasons could be that the expression of other targets such as *STP13*, *SBT* might be affected by miR394b and not *FBX*.

In our study, ABC transporter C family member 8-like and acetate/butyrate CoA ligase AAE7 were identified as targets of miR477a-3p. The up-regulation of these genes in the cold tolerant genotype (H) was compared to that of the cold sensitive almond varieties (Sh12) in both tissues. The ABCC multidrug resistance associated proteins (ABCC-MRP), a subclass of ABC transporters, is involved in multiple physiological processes such as cellular homeostasis and metal detoxification [69]. The genes belonging to this subfamily were identified in various species [70]. For instance they have 120 members in *Arabidopsis thaliana* and rice (*Oryza sativa*) [71]. Most ABC proteins, as membrane proteins, mediate MgATP-energized trans-membrane transport and/or regulate other transporters. These proteins are recognized as the key manager for plants under abiotic and biotic stresses [72]. The identified roles of this group of proteins include polar auxin transport, lipid catabolism, xenobiotic detoxification through transporting toxic compounds from cytosol into the vacuole [73] disease resistance, and stomatal function [71]. The induction of this ABC transporter in cold-tolerant tomato cultivar has been reported [37]. In our study, the significant up-regulation of ABC transporter in the H genotype, and not in the Sh12 genotype, revealed the involvement of this gene in cold tolerance, possibly through different processes such as detoxification mechanisms.

Acetate/butyrateCoA ligase AAEs, the other miR477a-3p target, participates in a variety of anabolic and catabolic pathways. Most AAEs share conserved structural elements such as a motif, which is necessary for binding to ATP and adenylate formation [73]. AAE7 show high expression level in many tissues including root, leaf, stem, flowers and the developing seeds [74]. Allen and colleagues [75] found the relationship between glutamine level and AAE7 accumulation. In their study, mutations in AAE7 or ACN1 (acetate non-utilizing 1) gene led to nearly 50% decrease in 13C-labelling of glutamine, which is a major carbon sink in seedlings. Glutamine (Gln) is a major nitrogen source in plants and is a central intermediate for carbon-nitrogen assembly. Glutamine synthetases (GS) was employed to maintain a sufficient Gln supply. This enzyme has cytosolic GS1 and plastidic GS2 for Gln production. Ji [76] showed that GS1 is responsive to various environmental stresses and also the involvement of GS1s in Gln homeostasis. For instance the activity of *GS1* in tomato was increased after exposure to different NaCl concentrations [77]. Two-dimensional (2D) gel electrophoresis analysis of proteins revealed that GS1 accumulates in response to cold treatment [78]. According to these findings and the induction of AAE7 under cold stress in the current study, it can be concluded that miR477a-3p, via its regulatory role on AAE7, could prevents the reduction of Gln supply, which is essential for growth and developments.

## Conclusion

Over the last decade, miRNAs have emerged as playing a major role in plants’ responses to different stress conditions. This is especially true by employing advanced NGS technologies, which have significantly increased the number of identified miRNAs in several plant species. In this study, we have outlined the current status of miRNA research in almond as an economically important fruit tree. Although miRNA research in fruit trees has mostly been descriptive to date, miRNAs offer great potential for the trait improvement of these crops. Hundreds of diverse miRNA genes including highly conserved, non-conserved and several species-specific miRNAs have been identified in peach, apple, citrus and grapevine species. Putative targets for the identified miRNAs have been described in the literature and include homologues of well-characterized targets from model plant species. An important goal of this study was to identify target mRNAs for species-specific miRNAs, and to elucidate their associated functions. Although several experimental approaches have been used to demonstrate function of miRNAs in model species such as Arabidopsis, rice and poplar, these methods have not been readily applied to fruit trees and almond to date.

One of the areas of plant biology that could benefit from NGS study is the investigation of gene expression under stress. Small RNAs contents of plant tissues could be easily sequenced and analyzed to identify important miRNAs under stress. Since spring frost stress is the most important environmental constraints for almond, we have analyzed cold-stress-related miRNAs in almond at two levels (0°C and -2°C) of cold exposure by stem_loop qPCR. Furthermore, we retrieved the expression profile of predicted target genes from two almond genotypes, which were obtained from a public database. Different expression profiles of target genes were observed among varieties. Comparative analysis of the gene expression of the miRNAs and their related targets, between cold tolerant and sensitive varieties grown under the same condition, allowed us to identify responsive miRNAs that are associated with cold tolerance. It further allowed us to make distinction between different varieties of the same species. In this study miR162, miR166d, miR168, miR171a, miR398a-3p, miR403, miR482f, miR6285, miR8123-5p, miR403, miR1511-3p and miR7122a-3p were introduced as differentially expressed miRNAs between H and Sh12 genotype. Further studies such as overexpression and/or RNAi strategies are needed to elucidate the precise role of these genes in almond.

## Materials and Methods

### Plant materials and stress condition

*Prunus dulcis* Mill genotype H, a cold tolerant and late blooming plant (REF), was used for sRNA sequencing. Sh12, which is also a cold sensitive genotype, was used for expression pattern confirmation. Branches were collected in the popcorn stage during early spring. The cut end of branches were placed in a 5% sucrose solution and then subjected to 0°C for 3h, -1°C for 2 h and -2 for 1 h, consecutively. The temperatures were chosen based on previous studies of Miranda *et al*. [2] and Kodad *et al*. [79]. As control treatment, few branches were kept at 10°C. Collected samples (ovary and anther) were immediately placed in liquid nitrogen and later stored at -80°C.

### RNA isolation and sequencing

Total RNA was isolated from 200 mg of anther and ovary samples using the method described by Rubio-Piña and Zapata-Pérez [80]. In this protocol, after samples were powdered, 1 ml extraction buffer containing 2% (w/v) CTAB; 0.1 M Tris-HCl (pH 8); 1.4 M NaCl; 20 mM EDTA (pH 8); 2% (w/v) PVP and 70 μl of β-mercaptoethanol was added to each sample. Then they were incubated at 65°C for 10 min. After incubation 800 μl chloroform was added and centrifuged at 10,000 rpm for 10 min at 4°C. Two other centrifugations were done after 800 μl phenol/chloroform (1:1) and chloroform/isoamyl alcohol (24:1) were added in equal volume to the supernatant. Finally, the last step was to add LiCl (8M) and to do centrifugation. Formed RNA pellets were washed and after a short time for drying, dissolved with DEPC water. Extracted RNA were qualified and quantified after DNAaseI treatment by spectrophotometer and agarose gel electrophoresis. cDNA library construction and sequencing (Illumina HiSeq 2000 platform) were perfomed by Macrogen company (Korea). Data was produced in FASTQ format and was obtained from the company’s server. The raw sRNA data has been deposited in the sequence reads archive (SRA), NCBI, and could be accessed using Bioproject number PRJNA245549 and Biosample accession numbers SAMN04317490, SAMN04317478, SAMN04317221 and SAMN04317155.

### Identification of conserved miRNAs in almond

For identification and analysis of our reads we have followed the procedure undertaken by Barakat *et al*. [12] with some modifications as follows. After quality control using SolexaQA software [81], sequences between 18 and 24 were extracted using an in-house perl script. Using BLASTN program, sRNA sequences that passed the size filter were then queried against rfam (http://rfam.sanger.ac.uk/), chloroplast and mitochondrial genomes (http://gobase.bcm.umontreal.ca/), rRNA (http://lowelab.ucsc.edu/GtRNAdb/), tRNA (http://www.psb.ugent.be/rRNA/), snoRNA (http://bioinf.scri.sari.ac.uk/cgi-bin/plant_snorna/home/) and all contaminating rRNA, tRNA and snoRNA sequences were removed. To identify conserved miRNAs, unique sRNA sequences were blasted against known miRNAs (http://www.miRbase.org/index.shtml) using default parameters, allowing up to two mismatches.

### Identification of non-conserved, almond-specific miRNAs

Sequences with no similarity to known miRNA sequences were used to search for non-conserved miRNAs in almond. The genome of almond has not been sequencesd yet. In order to obtain the possible precurseors for miRNAs, therefore, we had to map small RNA sequences according to the almond RNA-seq assemblies provided by Mousavi *et al*. [3]. Bowtie software (version 2) was used to map the reads on the contigs [82]. Regions harboring sRNA hits (150 at each side) were then obtained using a shell script. Next, Vienna RNA package was used for predicting miRNAs secondary structure [83]. The structures were then checked for miRNA features using MiRCheck program. Sequences that passed MiRCheck were sorted for redundancy. The sRNAs that passed the MiRCheck filter were then manually folded using mFold and assessed for miRNAs structure.

### Differential expression analysis of miRNAs related to cold stress

The normalization of known miRNAs frequency in four libraries was calculated to earn transcripts per million (TPM) according to the formula:

Normalized expression=(preliminary miRNA count/ total clean reads count)×1,000,000.

If the abundance of normalized values was zero, it was modified to 0.01. The *p*-value and fold-change (log_2_ ratio (treat/control)) were calculated for differential expression analysis. The *p*-value was obtained according to Gao *et al*. [42] and Li *et al*. [84]. The criteria for significant up/down-regulation was that, log_2_ ratio (treat/control) > 0.5 and < −0.5, and the *p*-value of < 0.05.

### Target prediction for miRNA sequences

The target genes of miRNAs were identified using psRNATarget (http://plantgrn.noble.org/psRNATarget/). This server is widely used for plant miRNA target prediction [85]. In order to identify almond specific target genes, which are under control by conserved miRNAs, we have used almond contigs assembled by Mousavi *et al*. [3]. Appropriated BLASTN hits with the highest identity were selected as predicted targets.

### Confirmation of miRNAs and their potential target genes by qPCR

Sixteen known miRNAs with the distinct difference in expression in cold stress vs. control condition were selected for further confirmation. In addition to miRNAs, eight predicted target genes of six differentially expressed miRNAs (miR162, miR166d, miR394b, miR398b, miR477a-3p and miR7122a-5p) were selected to assess reverse expression pattern between miRNAs and their target genes. Relative expression of candidated miRNAs in the cold tolerant genotype (H) was compared with the sensitive variety (Sh12) in various thermal treatments (10 and -2°C). The first strand cDNA synthesis for each miRNA was performed according to Varkonye-Gasic *et al*. [86] method. In this protocol 2μg of extracted RNA was reverse transcribed to cDNA using M-MuLV Reverse Transcriptase and stem-loop RT-PCR miRNA primers. All these primers were designed with miRNA primer designer software. To evaluate the expression of target genes by qPCR, the first strand cDNA Was synthesized using oligo-dT primers. The qPCR reaction in a total volume of 12 μl was performed on a Roter-Gene Q (Qiagen). Eeach reaction contained 2 μl of cDNA, 6 μl SYBR Premix Ex Taq (Takara) and, 0.83μM of forward and reversed primers. Three technical replicates were included for each biological repeats. The PCR amplification condition was performed according to Mousavi *et al*. [3]. miRNA specific primers, used in the current study, are in Table 2. For data normalization of miRNAs, 18S rRNA [87] was used, while PduAct1 [88] was applied as the internal control for target genes. The analysis was performed based on 2^−ΔΔCT^ method [89].

**Table 2:** The list of miRNA specific primers for confirmation by qPCR.

Primer name	miRNA Primer sequences (5' to 3')	Tm (°C)
Pdu-miR160a	GCG GCG GTG CCT GGC TCC CTG	73.1
Pdu-miR162	GCG GCG TCG ATA AAC CTC TGC	65.3
Pdu-miR166d	GCG GCG TCG GAC CAG GCT TC	68.7
Pdu-miR168	GCG GCG GTC GCT TGG TGC AGG TC	73.6
Pdu-miR171a	GCG GCG GTG ATT GAG CCG TGC C	71.5
Pdu-miR319a	GCG GCG GTT GGA CTG AAG GGA G	69.6
Pdu-miR394b	GCG GCG TTG GCA TTC TGT CCA C	67.7
Pdu-miR398a-3P	GCG GCG GTG TGT TCT CAG GTC G	69.6
Pdu-miR398b	GCG GCG CGT GTT CTC AGG TCG	69.2
Pdu-miR403	GCG GCG GTT AGA TTC ACG CAC	65.5
Pdu-miR477a-3P	GCG GCG GTT GGG GGC TCT TTT G	69.6
Pdu-miR482f	GCG GCG GTC TTT CCT ACT CCA C	67.7
Pdu-miR1511-3P	GCG GCG GAC CTG GCT CTG ATA C	69.6
Pdu-miR6285	GCG GCG GTA GTG AAG TTT GAA T	62.1
Pdu-miR7122a-5P	GCG GCG TTA TAC AAT GAA ATC	57.4
Pdu-miR8123-5P	GCG GCG GTG AGC AAT GGC ACA C	69.6
Universal reverse	ATC CAG TGC AGG GTC CGA GG	64.6

### Validation of direct target genes for selected miRNAs

2μg of extracted RNA from ovary tissue of H genotype under cold stress (-1°C) treatment was ligated to a 5′RACE DNA adaptor. Random nine-mer primers were then used for cDNA synthesis. Nested PCR was performed for amplification of a cDNA fragment, which contains the cleavage site of the targets. The first PCR reaction was done using the 5′RACE outer primer and a gene-specific outer primer. The second PCR was performed using the outer PCR reaction product as template, the 5′RACE inner primer and gene-specific inner primer (Table 3). PCR products were purified using the Gene JET^™^ Gel Extraction kit according to the manufacturer's recommendations (Fermentase) and were sequenced by Macrogen Company (Korea) through Sanger sequencing.

**Table 3:** The lists of primers for confirmation of predicted miRNA target genes.

Primer- Adaptor	5´to 3´The sequence of primer	Tm (°C)
Pdu-*Fbox*- RACE Outer Primer	TTCAAGCCACAGCAGTCA	60.5
Pdu-*Fbox*- RACE Inner Primer	CCACACAACCAGGGACCTTC	62.5
Pdu-*Fbox*- RACE Forward Primer	TGGAAAGCACGTAAGGATGACT	60.3
Pdu-*DCL*- RACE Outer Primer	GTACGCTCATTTGTGCCAAGG	62.1
Pdu-*DCL*- RACE Inner Primer	CACCGACCACAGGAACAAACA	61.3
Pdu-*DCL*- RACE Forward Primer	TCGCTCGAACCATGACTACAA	59.4

## Supporting Information

S1 Table Conserved miRNAs from reproductive tissues of almond. (XLSX)

S2 Table Novel miRNAs from reproductive tissues of almond. (XLSX)

S3 Table Profile expression of conserved miRNAs. (XLSX)

S4 Table The predicted targets for some cold-responsive conserved miRNAs. (XLSX)
